# Implementing a novel PAM-4 modulation/demodulation scheme along with source and link protection in a high capacity data center architecture

**DOI:** 10.1371/journal.pone.0341605

**Published:** 2026-03-13

**Authors:** Baozhu Li, Salman Ghafoor, Jawad Mirza, Bilal Aziz, Muhammad Ijaz, Ahmad Almogren

**Affiliations:** 1 School of Computer Science, Huanggang Normal University, Huanggang, China; 2 SEECS, National University of Sciences and Technology, Islamabad, Pakistan; 3 SEECS Photonics Research Group, Islamabad, Pakistan; 4 Telecommunication Department, Hazara University (HU), Mansehra, Pakistan; 5 Department of Engineering, Faculty of Science and Engineering, Manchester Metropolitan University, Manchester, United Kingdom; 6 Department of Computer Science, College of Computer and Information Sciences, King Saud University, Riyadh, Saudi Arabia; Manipal Academy of Higher Education, INDIA

## Abstract

Desirable data-center features include cost efficiency, high bandwidth, scalability, and protection against component failures. In this study, we propose an architecture that employs a small number of optical sources to transport data over a large number of links inside a data center. To further increase the bandwidth, two bits per symbol are transmitted by employing a novel technique for the generation and demodulation of pulse amplitude modulated (PAM) signals having a data rate of 100 Gbps. The proposed architecture provides protection against two major sources of breakdown, the laser source and the channel used for the transmission of signals. Furthermore, diversity gain can also be achieved by simultaneously transmitting the same optical signals through different channels inside the data center. The performance of the proposed architecture is observed in terms of bit error rate under different amplified spontaneous emission (ASE) noise powers.

## 1. Introduction

Data center networks (DCNs) play a crucial role in driving the increasing demand for optical communication capacity [1]. A significant portion of global DCN traffic is generated by intra-data center communications, which are predominantly associated with activities such as data backup, replication, read/write operations, and the extensive application of parallel computing across distributed processing and storage nodes [2]. Traditional fiber-based DCNs, which rely on multi-tier switch architectures, often face significant challenges during installation and operation due to the complexity of extensive cabling. This complexity leads to longer installation times and problems such as lossy or contaminated connectors, elevated power consumption, network oversubscription, limited scalability, and physical problems like micro-bends and fiber fractures, which can lead to system outages or network failures [3].

**Fig 1:**
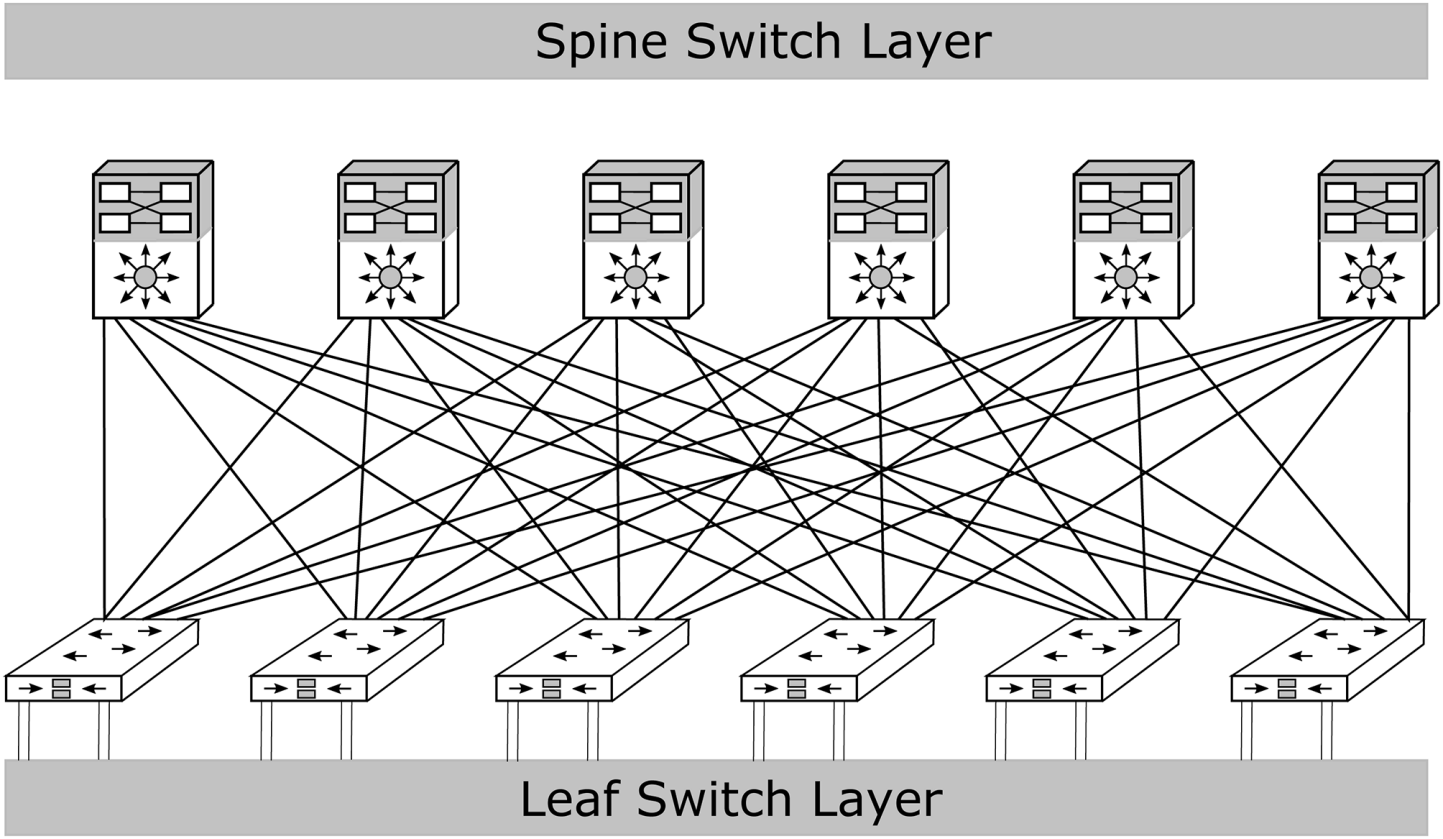
Spine-leaf data center architecture utilizing fiber-based links.

The spine-leaf architecture, a common DCN design utilizing optical fiber as the transmission medium, has been widely adopted in numerous DCNs, such as those by Cisco [4] and Meta [5]. As depicted in Fig 1, the spine-leaf architecture comprises two layers of switches: the spine layer and the leaf layer. The leaf layer manages traffic from servers, while the spine layer connects all leaf switches in a bipartite fashion [6]. This architecture is characterized by its two primary components—the spine and leaf-switching layers. One of its key advantages is that each leaf switch is connected to every spine switch within a pod, significantly enhancing communication efficiency and minimizing server-to-server latency. Moreover, the spine-leaf architecture eliminates the need for costly core-layer switches and facilitates incremental expansion by allowing additional switches and devices to be seamlessly integrated as business demands grow, thus optimizing initial investment costs.

Recent research on incorporating Free Space Optics (FSO) technology into DCNs has gained considerable interest [7]. FSO is an optical communication method that primarily uses coherent narrow-beam laser diodes as light sources and photodetectors as receivers to create highly directional line-of-sight (LOS) links between transceivers, facilitating long-distance, point-to-point transmissions [8]. In comparison to RF technology, indoor FSO offers several advantages for DCN applications, including lower power consumption, unlicensed operation, smaller antenna size, resistance to interference and multi-path fading, enhanced security due to the inability to penetrate obstacles, higher data rates (ranging from Gbps to Tbps), and minimal path loss (approximately 0 dB over 15 meters) [9]. Compared to optical fibers, FSO provides similar bandwidth, shorter network installation times, faster propagation speeds (approximately 1.5 times faster), and inherent immunity to issues such as dispersion, birefringence, fiber non-linearities, micro-bends, and fractures. These advantages drive the motivation to integrate FSO technology into DCNs [10]. Hybrid FSO/fiber-based designs are also being investigated. For example, Liu et al. [3] proposed a hybrid beam-steering free-space and fiber-based optical data center network, where the main objective was to enhance topological flexibility and bandwidth scalability through active beam alignment and reconfigurable optical paths. Their architecture integrates micro-electro-mechanical system (MEMS) mirrors and free-space transceivers for dynamic inter-rack connections, providing an adaptable physical layer that mitigates the limitations of fixed fiber links.

A critical element of data center operations is redundancy, which protects against potential component failures, such as optical source or channel disruptions [11]. Redundancy is essential because even minimal interruptions in data center services can have severe financial consequences, with downtime leading to significant monetary losses. Redundancy models are especially important because many data centers incorporate uptime guarantees within their service level agreements (SLAs), and data center clients often extend these guarantees to their end users. Without dependable data center operations and consistent uptime, the financial impact of downtime can be overwhelming. Moreover, business continuity is heavily dependent on maintaining uptime, which is greatly supported by redundant systems [11]. By proactively planning for component failures and implementing redundancies, data centers can enhance uptime and by extension, ensure business continuity. Another pressing challenge for data centers is the occurrence of micro-bursts—intense spikes in traffic that occur over short durations. In recent years, both the academic and industry communities have increasingly focused on addressing micro-bursts in modern data centers, as these traffic surges can result in serious performance issues [12].

Several research efforts have explored PAM-4 modulation to enhance data rate and spectral efficiency in optical interconnects for DCNs. For example, Verbist et al. demonstrated a DAC-less and DSP-free 112 Gb/s PAM-4 transmitter using two parallel electroabsorption modulators (EAMs) driven by decorrelated electrical signals, thereby eliminating the need for high-speed digital processing [13]. Similarly, Chao et al. present a single-channel 112 Gbps PAM-4 transmission over 1 km SMF using IM/DD; uses a low-cost directly modulated laser with 18-GHz bandwidth [14]. In [15], A DAC-less PAM-4 signal generation technique has been demonstrated using a silicon Mach–Zehnder interferometer (MZI) modulator configured in a series push–pull arrangement. In this approach, two independent binary electrical signals with different amplitudes are applied to the modulator arms, effectively generating the four-level optical waveform. A 50 Gbps per-wavelength PAM-4-based passive optical network (PON) has been experimentally demonstrated using a 10G-class receiver. To mitigate channel distortions, a memory polynomial equalizer (MPE) combined with a decision feedback equalizer (DFE) was employed [16].

Based on the discussion above, it can be concluded that the generally desirable features for a data center architecture are high bandwidth, protection against component breakdown, scalability and cost-efficiency. In this study, we propose a novel data center architecture to achieve some of these desirable features. The novel contributions of our work are as follows:

- To cope with the large number of connections between the spine and leaf layers of the DCN, we have generated optical combs with a large number of optical sidebands, where each sideband can be individually modulated for data transmission.
- A novel technique for all-optical implementation of pulse amplitude modulation (PAM-4) and demodulation has been proposed. PAM-4 enables us to achieve higher data rates by transmitting two bits per symbol with relatively less complexity.
- Our proposed architecture provides protection against the breakdown of the transmission channel and the optical source. In case one comb source breaks down, the proposed technique allows us to automatically switch to the second comb. The transmission channel is composed of a hybrid fiber/FSO link. In case one of the link is not available, the data is automatically transmitted over the other.
- Finally, our proposed technique allows us to achieve diversity gain by simultaneously transmitting the same data over both the fiber and FSO links. This also results in increased reliability, since the two transmission media experience different types of impairments, the chances of both being affected simultaneously are significantly reduced.

## 2. The proposed PAM-4 modulation/demodulation technique

**Fig 2:**
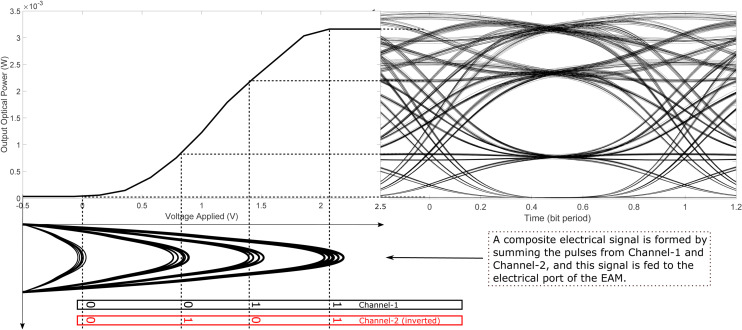
Optical PAM-4 signal synthesis from two binary inputs using an EAM.

To generate an optical PAM-4 signal from the data streams of Channel-1 and Channel-2, the transfer function of an EAM is utilized, as illustrated in Fig 2. The electrical pulses corresponding to the two channels are linearly superimposed to produce a single composite electrical signal whose amplitude equals the sum of the individual pulses. Importantly, the peak amplitudes representing logical ‘1’ and ‘0’ are assigned distinct values for each channel. This differentiation enables the composite signal to produce unique amplitude levels for the bit combinations ‘01’ and ‘10’, as depicted in Fig 2. The resulting electrical signal is then applied to the electrical input of the EAM, while a continuous-wave optical signal is fed into its optical input. Due to the nonlinear transfer characteristic of the EAM, variations in the input voltage lead to corresponding changes in the optical output power. Since the input electrical signal assumes four discrete amplitude levels, the modulated optical output exhibits four distinct intensity levels, corresponding to the bit combinations ‘00’, ‘01’, ‘10’ and ‘11’, as shown in Fig 2. It is noteworthy that the data bits from Channel-2 are inverted prior to their summation with Channel-1. The rationale behind this inversion will be clarified in the demodulation discussion that follows. In summary, this approach combines the data from two binary channels into a single PAM-4 optical signal, effectively encoding two bits per symbol.

**Fig 3:**
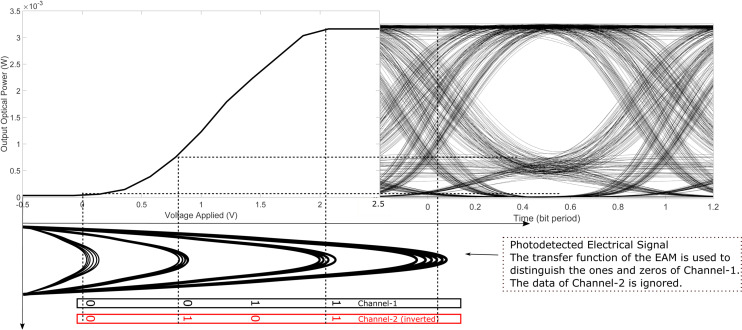
Demodulation of Channel-1 data by employing the transfer function of an EAM.

We now present the proposed demodulation technique for recovering the data bits of Channel-1 and Channel-2 from the received PAM-4 signal. The method for extracting Channel-1 data is illustrated in Fig 3. Demodulation is carried out using an EAM at the receiver, which has a transfer function similar to that of the EAM used at the transmitter for PAM-4 signal generation. The received optical PAM-4 signal is first photodetected and then passed through a low-pass filter to recover the corresponding electrical PAM-4 waveform.

A separate optical source is connected to the optical input of the receiver-side EAM. As described in the following section, this optical carrier is transmitted alongside the PAM-4 data signal from the transmitter. The average power level of the electrical PAM-4 signal is chosen such that the lower-amplitude levels corresponding to bit combinations ‘00’ and ‘01’ fall within the high-attenuation region of the EAM’s transfer function, resulting in very low output optical power, as shown in Fig 3. In contrast, the higher-amplitude levels representing ‘10’ and ‘11’ are located in the low-attenuation region, allowing greater transmission of optical power.

This selective attenuation enables the recovery of Channel-1 data by effectively distinguishing between logical ‘0’ and ‘1’ based on the output optical power. The contribution from Channel-2 is suppressed in this step and will be recovered separately using an additional demodulation path, as described later. It can also be observed in Fig 3 that the nonlinear (compressive) nature of the EAM’s transfer function reduces the amplitude variation of the higher-level electrical signals. This compressive behavior mitigates amplitude distortions induced by the optical channel, thereby improving signal robustness.

**Fig 4:**
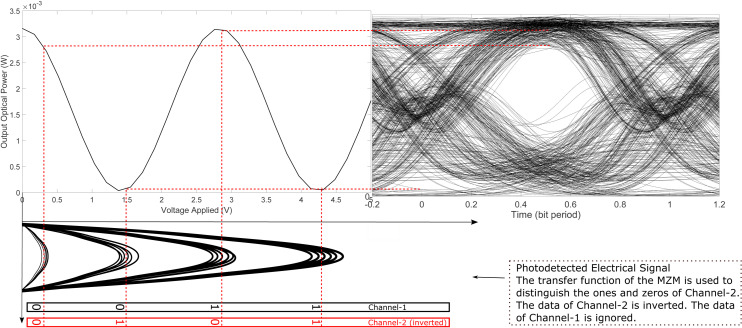
Demodulation of Channel-2 data by employing the transfer function of an MZM.

Finally, we describe the demodulation technique employed to recover the data bits of Channel-2. In this case, a Mach-Zehnder Modulator (MZM) is used, leveraging its transfer function as depicted in Fig 4. Analogous to the demodulation approach for Channel-1, a portion of the received optical PAM-4 signal is photodetected to obtain the corresponding electrical signal. The signal amplitude is then adjusted such that the levels corresponding to the bit combinations ‘00’ and ‘10’ fall within the low-attenuation region of the MZM transfer function, while those corresponding to ‘01’ and ‘11’ are mapped to the high-attenuation region, as shown in Fig 4.

A dedicated optical carrier, transmitted from the source alongside the data, is fed into the optical input of the MZM. Through this mapping, the MZM produces an output optical signal with higher power for input bits ‘00’ and ‘10’ and lower power for ‘01’ and ‘11’. Consequently, the output optical power is high for logical ‘1’ bits and low for logical ‘0’ bits of Channel-2. However, due to the nature of the MZM transfer function, this process inherently results in inversion of the logical levels. To correct for this inversion, the data bits of Channel-2 were intentionally inverted during modulation at the transmitter, as previously discussed. It is important to note that the information corresponding to Channel-1 is disregarded in this step, as it is demodulated in parallel using the EAM-based technique.

## 3. The proposed architecture

This section discusses the working principle of the overall architecture proposed in this work. The working principle is divided into three main parts, which include the optical frequency comb generation, the transmitter side, representing the leaf switch layer and the receiver side, representing the spine switch layer. These parts are discussed in the following sections.

### 3.1. Optical Frequency Comb Generation

**Fig 5:**
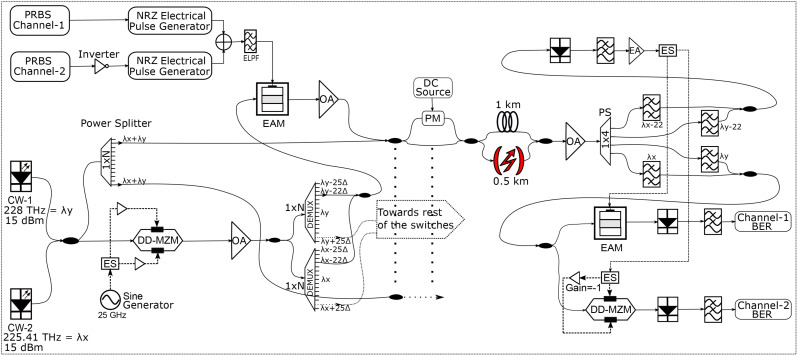
Simulation setup of our proposed PAM-4 based data center architecture.

Figure 5 shows the complete simulation design of our proposed technique. First of all, two laser sources centerd at 225.41 THz and 228 THz are used to simultaneously generate two frequency combs using a dual-drive MZM (DD-MZM). The multiple wavelengths of the frequency combs are used to connect multiple switches of the leaf and spine layers, as shown in Fig 1. The comb generation may be understood from the mathematical expressions for the DD-MZM. The input optical source to the DD-MZM can be represented by the following expression:


$$E_{in}(t)=\sqrt{E_{c}}\exp(j2\pi f_{c}t)$$ (1)

In the above expression, $E_{c}$ and $f_{c}$ represent the amplitude and center frequency of the input optical carrier. A sinusoidal signal is applied to the electrical input of the DD-MZM to generate multiple sidebands from each of the two laser sources connected to the optical input of the DD-MZM, as shown in Fig 5. The sinusoidal signal having frequency $f_{rf}$ = 25 GHz and amplitude of $V_{rf}$ is divided into two parts represented by $V_{1}$ and $V_{2}$. To operate the DD-MZM in a push-pull configuration, the polarity of $V_{1}$ is inverted relative to $V_{2}$. The signals $V_{1}$ and $V_{2}$ are represented by the following expression:


$$V_{1}(t)=\frac{1}{2}\{V_{rf}\sin(2\pi f_{rf}t)+V_{dc}\}=-V_{2}(t)$$ (2)

$V_{dc}$ in the above expression is the DC bias voltage of the DD-MZM. With the above-mentioned signals applied to the DD-MZM, the optical signal at the output $E_{out}(t)$ can be written as follows:


$$E_{out}(t)=\frac{E_{in}(t)}{2}[\exp\{j\frac{\left(1+\gamma_{c})\pi V_{1}(t\right)}{V_{\pi }}\}+\exp\{j\frac{\left(1-\gamma_{c})\pi V_{2}(t\right)}{V_{\pi }}\}]$$ (3)

$V_{\pi }$ is a constant and represents the voltage required to introduce a phase shift of *π* between the arms of the modulators. The parameter $\gamma_{c}$ represents the chirp parameter of the modulator.

By substituting the voltage values from Eq. 2 into Eq. 3 and simplifying gives us the following expression for the electrical field of the output optical signal:


$$E_{out}(t)=\frac{E_{in}(t)}{2}[\exp\{j\phi_{2}\sin(2\pi f_{rf}t)\}\exp(j\phi_{1})\;\;+\exp\{-j\phi_{4}\sin(2\pi f_{rf}t)\}\exp(-j\phi_{3})].$$ (4)

In the above expression, $\phi_{1}$, $\phi_{2}$, $\phi_{3}$ and $\phi_{4}$ are related to $V_{dc}$, $V_{rf}$ and $V_{\pi }$ through the following expressions:


$$\phi_{1}=\frac{\pi (1+\gamma_{c})}{2V_{\pi }}V_{dc},\;\phi_{2}=\frac{\pi (1+\gamma_{c})}{2V_{\pi }}V_{rf},\;\phi_{3}=\frac{\pi (1-\gamma_{c})}{2V_{\pi }}V_{dc},\;\phi_{4}=\frac{\pi (1-\gamma_{c})}{2V_{\pi }}V_{rf}.$$ (5)

The DD-MZM is biased such that it operates at the quadrature point on the transfer function. This can be achieved by making both $V_{dc}$ = $V_{\pi }$/2 and $V_{rf}$ = $V_{\pi }$/2. This implies that the peak-to-peak voltage of the sinusoidal signal is equal to $V_{\pi }$. By substituting these values of $V_{dc}$ and $V_{rf}$ in Eq. 6, we get $\phi_{1}$ = $\phi_{2}$ and $\phi_{3}$ = $\phi_{4}$ = *π*/2 – $\phi_{1}$. Using these values in Eq. 4 results in the following expression for the output of the DD-MZM in terms of $\phi_{1}$:


$$E_{out}(t)=\frac{E_{in}(t)}{2}\exp(j\phi_{1})[\exp\{j\phi_{1}\text{sin}(\omega_{rf}t)\}\;-j\exp\{(-j(\frac{\pi }{2}-\phi_{1})\text{sin}(\omega_{rf}t))\}]$$ (6)

Since sinusoidal intensity modulation is applied to the optical signal, the above expression can be represented in terms of Bessel function as follows:


$$\begin{array}{cc} & E_{out}(t)=\frac{\sqrt{E_{c}}\exp(j\phi_{1})}{2}[\{J_{o}(\phi_{1})-jJ_{o}(\phi_{1}-\frac{\pi }{2})\}\exp(j2\pi f_{c}t)\\ & \;+\sum_{n=-\infty }^{\infty }J_{n}(\phi_{1})\exp(j2\pi (f_{c}+nf_{rf})t)\\ & \;-j\sum_{n=-\infty }^{\infty }J_{n}(\phi_{1}-\frac{\pi }{2})\exp(j2\pi (f_{c}+nf_{rf})t)] \end{array}$$ (7)

**Fig 6:**
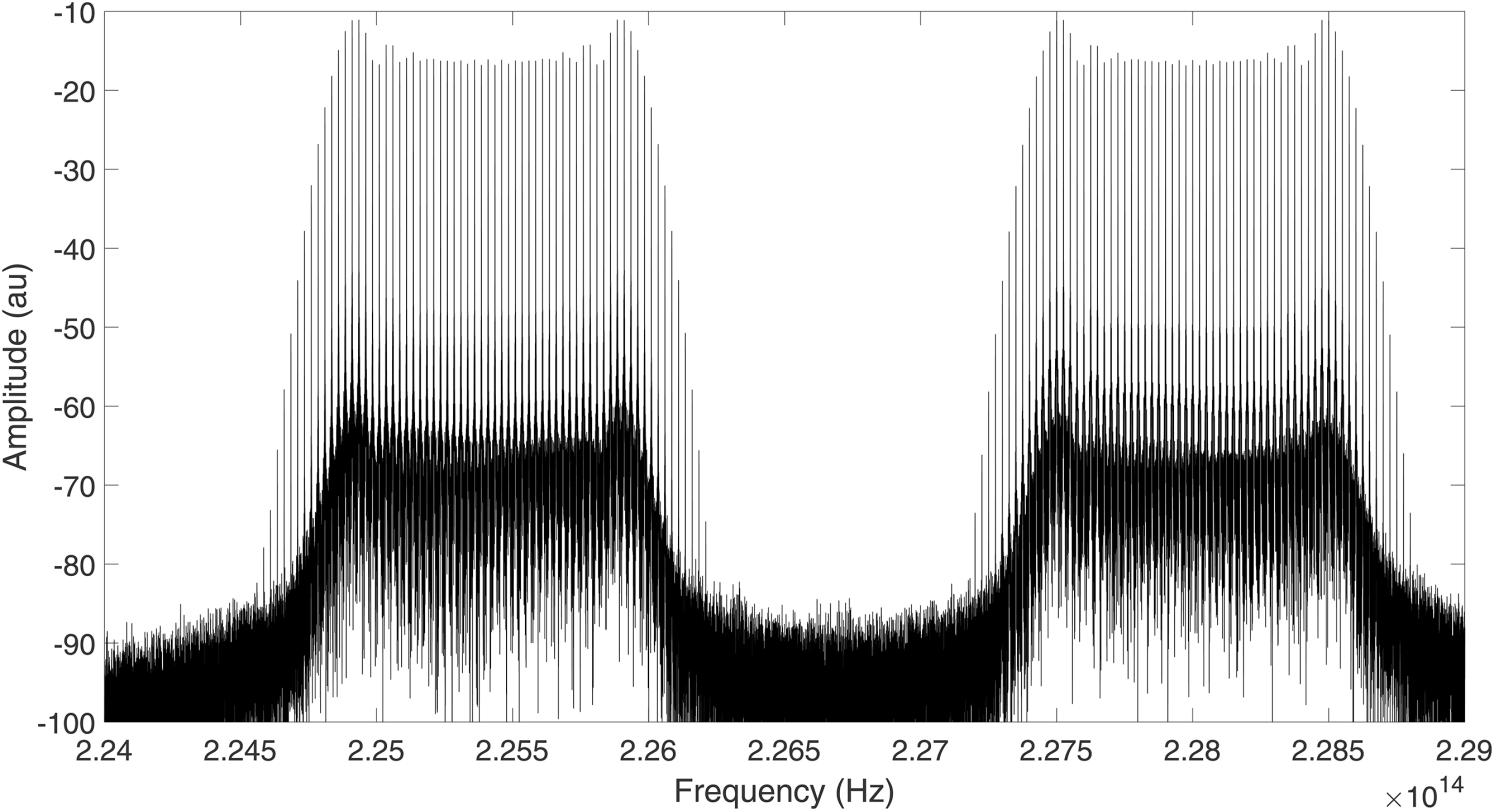
Two optical comb signals obtained at the output of the DD-MZM, centerd at 225.41 THz and 228 THz, respectively.

In the above equation, $J_{n}(\phi_{1})$ represents the $n^{th}$-order Bessel function. It may be observed that the output of the DD-MZM is composed of multiple sidebands, whose amplitudes are represented by Bessel functions of different orders. By increasing the value of $\phi_{1}$, we can increase the number of sidebands generated, hence resulting in an optical comb spectrum. Note that Eq. 7 represents the output of the DD-MZM when a single optical signal is connected to the input. In our simulation, two optical signals centerd at 225.41 THz and 228 THz are combined and connected to the optical input of the DD-MZM. Therefore, we obtain two separate optical combs centerd at these frequencies, as shown in Fig 6. It may be observed from Fig 6 that the comb spectrum is generally flat. We will use these multiple sidebands as separate optical carriers for transmitting a large amount of data between the switches of the spine-leaf architecture.

### 3.2. Transmitter Side

The transmitter side of the proposed architecture represents the spine switch layer of the DCN. Optical sidebands from each comb are used to transmit the data from each switch of the spine layer to the respective switch of the leaf layer. As shown in Fig 5, a 2×2 optical coupler is used to combine the output of the two laser sources centerd at 225.41 THz and 228 THz. Between the two outputs of the coupler, one is connected to the optical input of the DD-MZM for comb generation, while the other is connected to a 1×50 power divider. The comb signals at the output of the DD-MZM are amplified and connected to two separate 1×50 demultiplexers. One of the demultiplexers is centerd at 225.41 THz, while the other is centerd at 228 THz. The channel spacing of the demultiplexers is 25 GHz, which is equal to the spacing between adjacent sidebands of the comb. As shown in Fig 5, one sideband of each comb at the output of the demultiplexer is combined and connected to the EAM. PAM-4 modulation discussed in Section. 2, is applied to the combined optical signal through the EAM. The total data rate of the PAM-4 signal is 100 Gbps, representing a baud rate of 50 Gbaud per second. The PAM-4 modulated signal is amplified using an optical amplifier and combined with the unmodulated carriers obtained at the 1×50 power splitter output. This configuration at the transmitter side allows us to protect the link from laser source failure as well as transmit a large data between the spine and leaf layers of the DCN. Since the sidebands of both the laser sources are coupled in every link, if one of the lasers fails, the other will still enable us to transmit the data. The unmodulated optical signals are coupled with the PAM-4 modulated signals so that they may be used as optical inputs to the EAM and MZM at the demodulation stage at the receiver, as discussed in Section. 2. In Fig 5, we have shown the transmission of a single data channel by using the sideband number 22, which is located towards the lower wavelength or higher frequency side of the comb spectrum. The comb generation is discussed in Section. 3.1 gives us around 50 sidebands from each laser source. Similar to sideband number 22, the remaining sidebands can also be used simultaneously to transmit the data between the spine and leaf switches of the DCN.

Although only two comb lines are employed in this proof-of-concept simulation for clarity, the optical comb source inherently supports a large number of equidistant sidebands that can each serve as independent WDM channels. The proposed architecture can thus be scaled to tens of wavelengths for high-capacity data center networks. While the present study uses 25 GHz channel spacing for compatibility with standard DCN channel grids, the proposed architecture is adaptable to wider spacings (50–100 GHz) for higher symbol-rate operation. Such reconfigurability enables the system to evolve toward next-generation 100–200 Gbps-per-wavelength DCI requirements.

### 3.3. Channel between the Spine and Leaf Layers

The combined signal of two modulated optical sidebands and two unmodulated optical sources is transmitted to the respective switch through two different links. As shown in Fig 5, one of the link is based upon an FSO channel, while the other is based upon a standard single-mode fiber (SMF). A cross-coupler having a phase modulator (PM) is used to choose between the FSO or the SMF channel [17]. The electrical input of the PM is connected to a DC source. With no DC voltage applied, the combined optical signal exits through the lower output port of the coupler that is connected to the FSO link. When a constant DC voltage is applied, the combined optical signal exits through the upper port of the coupler that is connected to the SMF. The FSO channel has a length of 0.5 km while the SMF has a length of 1 km. These lengths are chosen to keep in view the maximum possible distances involved between the switches in a DCN. This arrangement protects the link against channel failures. For example, under normal conditions, the data may be transferred using the SMF link by applying a DC voltage to the PM. In case the SMF link breaks down due to any reason, the receiver will generate an alarm that may be used to automatically change the DC voltage to zero. As a result, the optical signal will now be routed seamlessly towards the FSO channel. Furthermore, if we choose an intermediate voltage, the signal will be split between the FSO and the fiber link. This simultaneous transmission of data towards the receiver will result in diversity gain and increased reliability of the link. The FSO channel model employed in our work is the log-normal model that represents medium to low turbulence [18], making it suitable to represent a data center scenario.

### 3.4. Receiver Side

The setup at the receiver should be such that it can demodulate the PAM-4 signal received through any of the two paths or any of the two lasers. As shown in Fig 5, the signal after transmission through the optical channel is amplified using an optical amplifier. The output of the amplifier is split into four parts using an optical power splitter. The optical signals centerd at 225.41 THz and 228 THz are filtered and combined using an optical combiner. These unmodulated optical signals are connected to the optical input of an MZM and EAM to perform PAM-4 demodulation using the principle discussed in Section. 2. The two modulated optical signals denoted as sideband number 22 from both the combs are also filtered and combined, as shown in Fig 5. It is important to mention here that at any one time, generally one of the laser sources will be operating. Therefore, we will have a single modulated and unmodulated signal at the receiver side. The modulated signals are photodetected and the resulting electrical signal is low-pass filtered and amplified. The amplified electrical signal is applied to the electrical input of the MZM and the EAM to perform PAM-4 demodulation. At the output of the MZM and the EAM, we will obtain two separate data streams representing channel-1 and channel-2, respectively. These signals are photodetected and low-pass filtered to obtain BER results.

Although two optical sources are employed in the simulation model for clarity, both originate from the same optical comb generator, which can be realized as part of a single multi-wavelength integrated laser array. In an integrated photonic platform, the demodulation structure can be compactly implemented using tunable couplers and on-chip optical phase shifters, significantly reducing complexity and cost. Furthermore, At high symbol rates, precise time and phase alignment between optical channels is critical. In practical hardware, this can be achieved using tunable optical delay lines or integrated phase shifters controlled via low-speed feedback loops. In the current simulation, ideal synchronization was assumed to isolate the modulation–demodulation behavior; however, real-time synchronization will form a key aspect of future experimental validation.

## 4. Results and discussion

**Fig 7:**
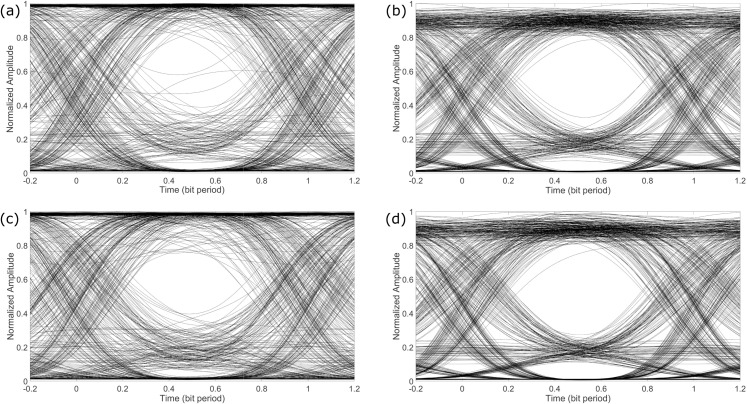
Eye diagrams for channel-1 for OSNR of 20 dB when transmitted (a) over fiber using a laser source of 225.41 THz, (b) over FSO using a laser source of 225.41 THz, (c) over fiber using laser source 228 THz and (d) over FSO using laser source 228 THz.

In this section, we discuss the results obtained for our proposed technique. Fig 7 shows the eye diagrams for channel-1 of the received PAM-4 signal transmitted over the fiber and FSO links using the two different laser sources centerd at 225.41 THz and 228 THz. Recall from Section 2 that channel-1 is demodulated using the EAM-based demodulator. These eye diagrams have been obtained by setting the optical signal-to-noise ratio (OSNR) of the PAM-4 signal equal to 20 dB. The eye diagrams of Fig 7 show that the channel-1 received through the FSO link has a better quality compared to the fiber link. The reason may be attributed towards the longer length of the fiber channel compared to the FSO link. Furthermore, the propagation of multiple wavelengths and noise through the fiber channel may result in some nonlinear interaction among these optical sources. As discussed in Section. 2, the EAM-based demodulation compresses the fluctuations induced over the pulses representing a one-bit. This compressive behaviour may be observed from the eye diagrams shown in Fig 7 and results in good signal quality. Due to this good quality of the demodulated signal, we can obtain an acceptable eye-opening even at a low OSNR of 20 dB.

**Fig 8:**
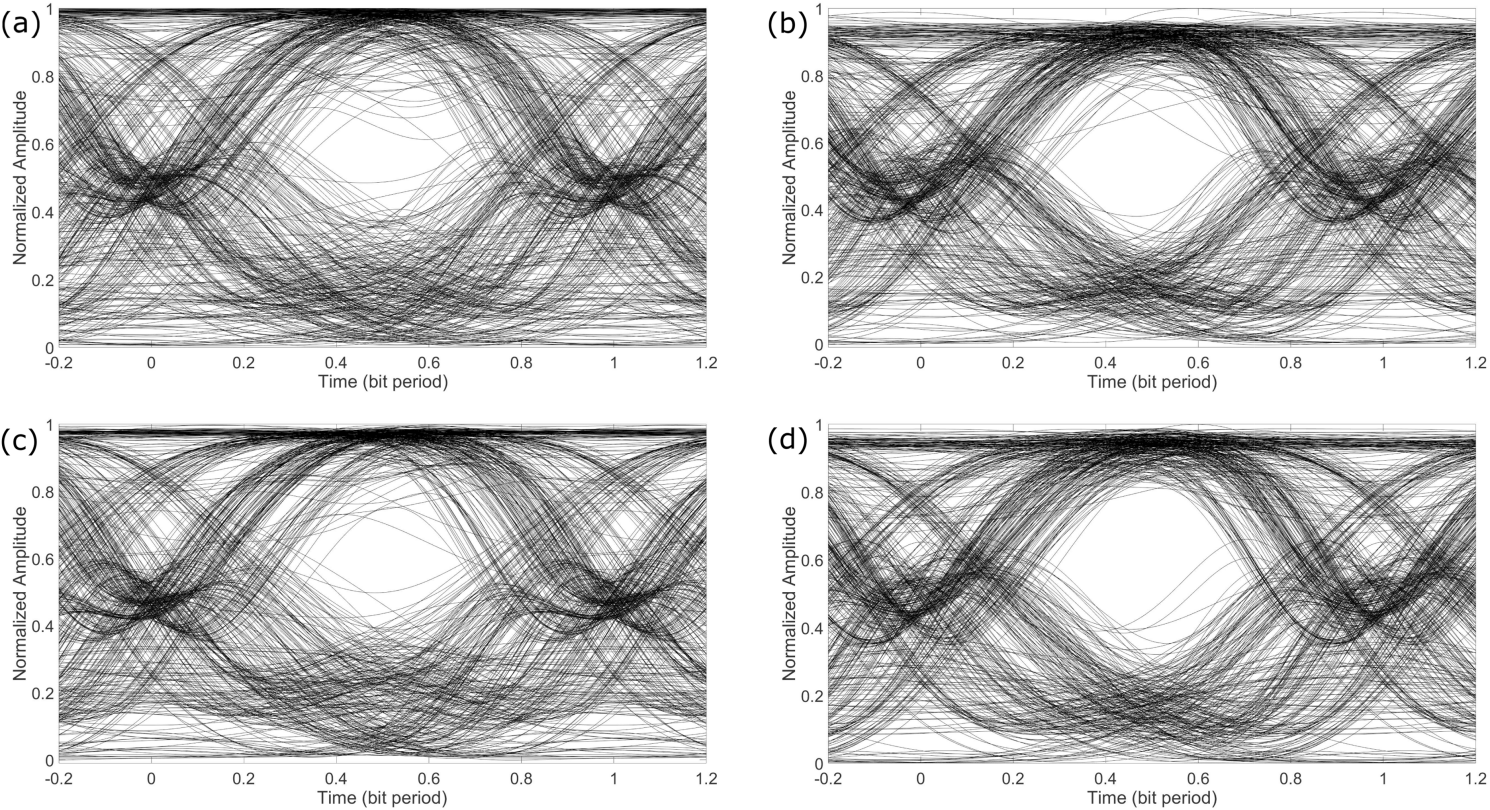
Eye diagrams for channel-2 for OSNR of 25 dB when transmitted (a) over fiber using a laser source of 225.41 THz, (b) over FSO using a laser source of 225.41 THz, (c) over fiber using laser source 228 THz and (d) over FSO using laser source 228 THz.

Fig 8 shows the eye diagrams of the demodulated channel-2 of the received PAM-4 signal. Since the quality of the demodulated channel-2 was not good, the eye diagrams have been obtained at a higher OSNR of 25 dB, compared to the eye diagrams of channel-1 shown in Fig 7. It may be observed from Fig 8 that even at higher OSNR, the eye-opening is not very wide, however, the receiver can still differentiate between a zero bit and a one bit. The reason for the lower performance of channel-2 may be understood from the way it is demodulated, as discussed in Section. 2 earlier. Channel-2 is demodulated using the multiple points on the transfer function of the MZM, as opposed to using only two points of the transfer function of the EAM. The four different amplitudes of the photo-detected electrical signal cannot be adjusted perfectly on the different points of the MZM transfer function, as shown in Fig 4. This discrepancy in adjusting the amplitudes results in intensity variations over the output optical pulses. The intensity variations are more pronounced for the signal transmitted over the fiber link, as may be observed from the eye diagrams in Fig 8a and c. Again, these pronounced intensity variations may be attributed towards the nonlinear interactions among the different wavelengths and optical noise travelling through the fiber. Overall, it may be observed that both channel-1 and channel-2 can easily be retrieved from the received optical PAM-4 signal.

**Fig 9:**
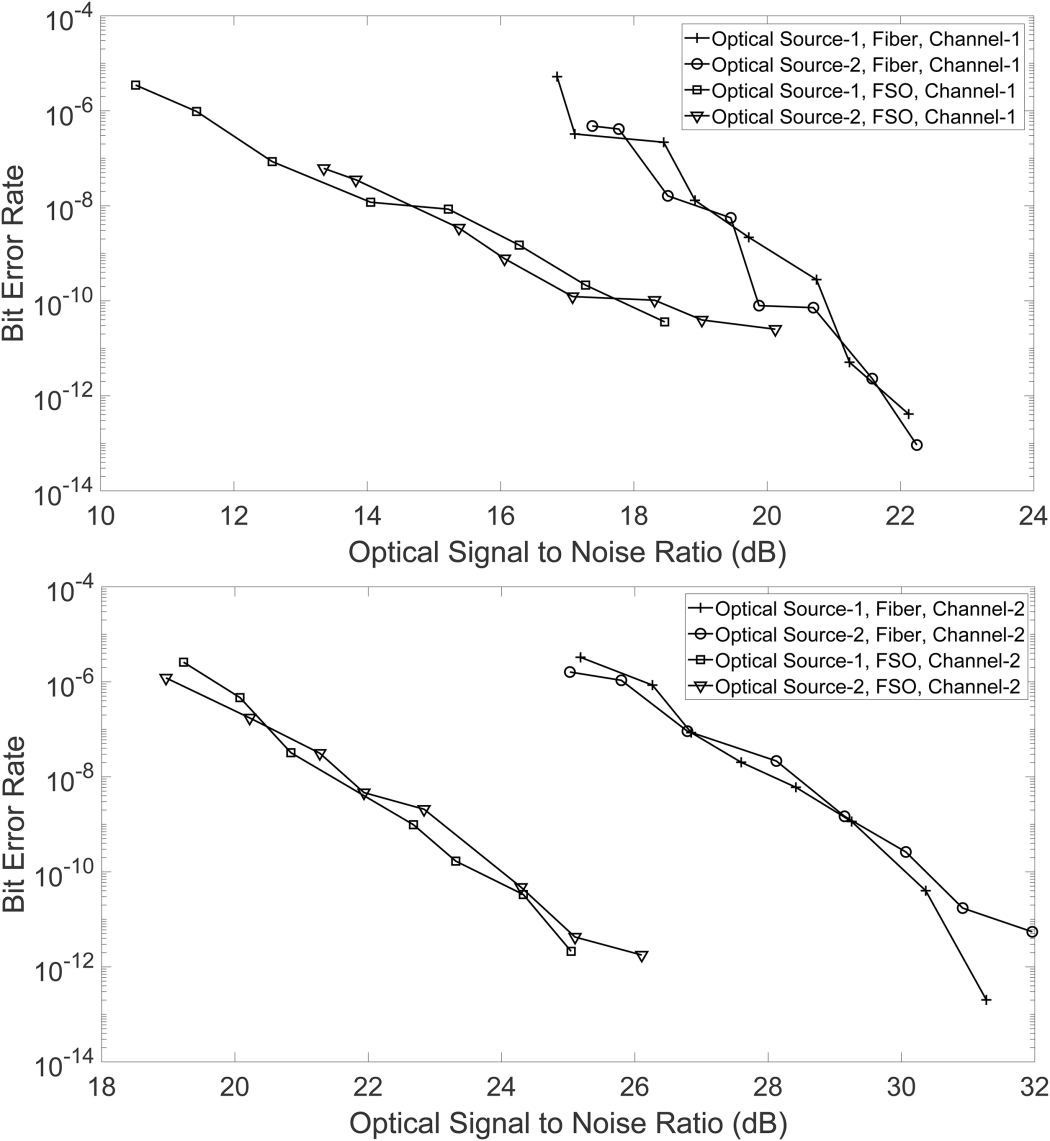
OSNR versus BER performance of the PAM-4 signal for (a) Channel-1 and (b) Channel-2. The results have been obtained for the two different optical sources and optical paths.

We have also obtained bit error rate (BER) results for the demodulated channels 1 and 2 for different values of the OSNR. The OSNR has been varied by adding inband amplified spontaneous emission (ASE) noise to the PAM-4 signal at the transmitter side. Fig 9a shows the BER versus OSNR plot for channel-1 when the PAM-4 signal is transmitted over the FSO and the fiber link. The plots have also been obtained for the two different optical sources centerd at 225.41 THz and 228 THz. The plot shows that channel-1 has a better BER when the optical PAM-4 signal is transmitted over the FSO link compared to the fiber link, for both the optical sources. Similar behaviour was observed from the eye diagrams discussed earlier. Furthermore, it may be seen that channel-1 has low BER values even for very low values of the OSNR. As discussed earlier, the reason for the better performance of channel-1 lies in the technique being used to demodulate it. As discussed in Section. 2, channel-1 is demodulated using only two points on the transfer function of the EAM. Therefore, it is relatively easy to adjust the PAM-4 signal amplitudes on the transfer function to extract the one and zero bits of channel-1. Another contribution to the better performance is from the compressive effect that the transfer function of the EAM induces over the intensity fluctuations of ones and zeros.

The BER versus OSNR performance for channel-2 is shown in Fig 9b. Generally, the BER of channel-2 is worse than channel-1 and hence requires higher values of OSNR to obtain the same range of BER values as channel-1. Again, the reason for this lower performance lies in the demodulation technique used to obtain the data bits of channel-2. As discussed in Section. 2, channel-2 is obtained by using four different points on the transfer of a MZM. Hence it is generally difficult to adjust the PAM-4 signal’s amplitudes on these four different points to obtain the zero and one bits of channel-2 from the received PAM-4 signal. As before, the BER performance is the same for both optical sources. However, the BER for the FSO link is better than that of the fiber link. This behaviour can be explained by observing that the length of the fiber link is twice that of the FSO link. Furthermore, the noise added at the transmitter side and the multiple optical signals co-propagating through the fiber may cause some nonlinear interactions among them. This nonlinearity results in higher amplitude fluctuations and timing jitter over the PAM-4 signal.

It is observed that the BER performance of the most significant bit (MSB) channel exhibits approximately an 8 dB power penalty relative to the least significant bit (LSB). This difference arises from the nonlinear transfer characteristics of the modulator, which result in unequal optical power spacing among PAM-4 levels. Similar asymmetries have been reported in other optical PAM-4 systems [13]. The penalty can be mitigated by employing bias pre-distortion or level equalization techniques to linearize the optical power transfer curve, which forms part of our future optimization work. It is recognized that the practical realization of an FSO-based DCI requires robust pointing, acquisition and tracking (PAT) mechanisms. In short-range inter-data-center deployments (typically up to a few hundred meters), compact micro-electro-mechanical (MEMS)-based beam-steering units and feedback-controlled optical transceivers have demonstrated high alignment accuracy, enabling stable operation under typical building vibration and thermal drift. While the present work focuses on demonstrating the feasibility and protection benefits of the hybrid fiber/FSO architecture, future studies will incorporate PAT modeling and adaptive control to assess the overall system robustness under realistic dynamic conditions.

While the proposed scheme maintains the same spectral efficiency as standard PAM-4 systems (i.e., 2 bits per symbol), variations in BER performance are attributed to differences in transmitter configuration, biasing approach, and optical-domain signal combination. The hybrid EAM–MZM-based architecture enables improved control over amplitude linearity and optical power levels across the four PAM levels, resulting in slightly better BER compared to purely EAM-based or MZM-based PAM-4 schemes. Table 1 summarizes this discussion.

**Table 1:** Comparison of the proposed PAM-4 modulation scheme with existing techniques.

Modulation format	Spectral efficiency (bits/s/Hz)	Typical BER (Simulated)	Architecture	Remarks
NRZ-OOK	1.0	$1\times 10^{-3}$	Single modulator	Limited data rate
Conventional PAM-4 (DSP-based)	2.0	$3\times 10^{-4}$	EAM or MZM + DSP	Requires digital equalization
Dual-EAM PAM-4 [13]	2.0	$2\times 10^{-4}$	Parallel EAMs	DSP-free, good linearity
Proposed Hybrid PAM-4 (EAM + MZM)	2.0	$6\times 10^{-5}$	Hybrid analog domain	Improved BER and optical redundancy

## 5. Conclusions

To cope with the increasing bandwidth demand of the data centers, we presented a comb-based architecture that enables the setting up of a large number of links between the switches of a DCN. Data transfer between the switches is based upon a novel PAM-4 modulation and demodulation technique that employs off-the-shelf components. In case the optical source and/or the optical channel breaks down, the backup source and/or the backup channel start working with minimal changes to the architecture. It was observed that the signals transmitted over the FSO channel performed better compared to the signals transmitted over the fiber link. Therefore, the FSO link may be used as the priority channel, while the fiber link may be used as a backup. It should be noted that by choosing a suitable phase shift of the phase modulator at the transmitter side, both channels can simultaneously transmit the same data, hence providing diversity gain. Eye diagrams for the received signals were presented under different OSNRs to understand how the PAM-4 demodulation is performed. Finally, we presented BER results to compare the performance of the architecture under different OSNRs. It was observed that the BER performance was satisfactory which allows for the architecture to be implemented in high-capacity data centers.

In comparison to conventional NRZ-OOK WDM systems, which offer simplicity but limited throughput, the proposed all-optical PAM-4 demodulation provides twice the data rate per channel while maintaining purely optical-domain processing. This approach minimizes electronic overhead and enhances scalability for future high-capacity data center networks.
